# A systematic review of the processes used to link clinical trial registrations to their published results

**DOI:** 10.1186/s13643-017-0518-3

**Published:** 2017-07-03

**Authors:** Rabia Bashir, Florence T. Bourgeois, Adam G. Dunn

**Affiliations:** 1 Centre for Health Informatics, Australian Institute of Health Innovation, Macquarie University, Sydney, NSW 2109 Australia; 2 Computational Health Informatics Program, Boston Children’s Hospital, Boston, MA USA; 3 Departments of Pediatrics and Emergency Medicine, Harvard Medical School, Boston, MA USA

**Keywords:** Clinical trials as topic, Trial registration, Publication bias, Reporting bias, Systematic reviews as topic

## Abstract

**Background:**

Studies measuring the completeness and consistency of trial registration and reporting rely on linking registries with bibliographic databases. In this systematic review, we quantified the processes used to identify these links.

**Methods:**

PubMed and Embase databases were searched from inception to May 2016 for studies linking trial registries with bibliographic databases. The processes used to establish these links were categorised as automatic when the registration identifier was available in the bibliographic database or publication, or manual when linkage required inference or contacting of trial investigators. The number of links identified by each process was extracted where available. Linear regression was used to determine whether the proportions of links available via automatic processes had increased over time.

**Results:**

In 43 studies that examined cohorts of registry entries, 24 used automatic and manual processes to find articles; 3 only automatic; and 11 only manual (5 did not specify). Twelve studies reported results for both manual and automatic processes and showed that a median of 23% (range from 13 to 42%) included automatic links to articles, while 17% (range from 5 to 42%) of registry entries required manual processes to find articles. There was no evidence that the proportion of registry entries with automatic links had increased (*R* ^2^ = 0.02, *p* = 0.36). In 39 studies that examined cohorts of articles, 21 used automatic and manual processes; 9 only automatic; and 2 only manual (7 did not specify). Sixteen studies reported numbers for automatic and manual processes and indicated that a median of 49% (range from 8 to 97%) of articles had automatic links to registry entries, and 10% (range from 0 to 28%) required manual processes to find registry entries. There was no evidence that the proportion of articles with automatic links to registry entries had increased (*R* ^2^ = 0.01, *p* = 0.73).

**Conclusions:**

The linkage of trial registries to their corresponding publications continues to require extensive manual processes. We did not find that the use of automatic linkage has increased over time. Further investigation is needed to inform approaches that will ensure publications are properly linked to trial registrations, thus enabling efficient monitoring of trial reporting.

**Electronic supplementary material:**

The online version of this article (doi:10.1186/s13643-017-0518-3) contains supplementary material, which is available to authorized users.

## Background

Clinical trial registries were established to improve transparency and completeness in the reporting of clinical trials [1–6]. Since they were established, a number of policies have been implemented to encourage or mandate their use, and this has led to substantial growth in the number of trials that have been registered [7–11]. For example, since 2005, prospective trial registration has been a condition for publication in member journals of the International Committee of Medical Journal Editors (ICMJE) [1, 12]. The European Union and USA have also passed legislation requiring prospective registration of clinical trials involving drugs or devices [13].

Clinical trial registries provide the ability to measure biases in the reporting of clinical trials that arise due to non-publication, delayed publication, or incomplete publication of results [14]. Studies examining these issues rely on the ability to establish a link between the original trial registration and subsequent published article. These links can be established in an automatic fashion if the publication abstract or metadata includes the registry identifier [15, 16]. However, if this identifier is not included by trial investigators or added by journals, manual processes are needed to create these links, either through searches and inference or through direct contact with investigators. Despite the number of studies that have examined reporting biases by linking trial registry entries and publications, the processes for linking are variable and poorly described.

Clinical trial registries are a critical source of information for systematic reviewers who use these registries to augment bibliographic database searches when compiling relevant evidence from clinical trials [17–19]. Systematic reviewers may seek to identify links from published trial reports to their respective registry entries to fill in gaps for information that is missing or incompletely reported. They may also independently search trial registries to identify additional trials [20, 21] and follow links from the registry to reports of the trials.

Our aim was to quantify the processes that have been used to link clinical trial registries with published results and to examine the use and utility of automatic linkage over time. To do this, we conducted a systematic review of all studies examining a cohort of clinical trials to identify links from clinical trial registries to bibliographic databases and from bibliographic databases to clinical trial registries, following a published systematic review protocol [22].

## Methods

### Inclusion criteria and search strategy

We identified all primary studies that examined links between any of the registries in the World Health Organization (WHO) International Clinical Trials Registry Platform (ICTRP) and published articles in bibliographic databases. Studies were excluded if there was no English-language version, if they did not unambiguously report the total number of clinical trials for which links were identified, if they were reporting on a specific clinical trial, or if the identification of links was not the primary focus of the study. Studies that did not unambiguously report the processes used to identify links were included in the review but excluded from the analyses.

PubMed and Embase were searched from inception to May 27, 2016, [23, 24]. The search strategy was developed with the assistance of a medical research librarian with details described in a previously published protocol [22]. The full version of the search strategy for both databases is provided in additional files (see Additional files 1 and 2). This strategy included searching of all study references to identify any other relevant articles not captured in the original search. Duplicate studies were removed using digital object identifiers and manually comparing titles, authors, publication dates, and article metadata. All identified studies were screened individually by two reviewers for inclusion, and disagreement was resolved through discussion.

### Data extraction

Two reviewers evaluated all the included studies to extract relevant information from the studies and resolved ambiguities by discussion. For each study, the following information was extracted: (a) number of reported clinical trials, (b) number of published articles, (c) trial registries used, (d) the study purpose (such as publication bias, outcome reporting bias, or assessing the publication rate of registered trials), (e) application domain (any constraints such as journal lists, conditions, or specialties), (f) processes for identifying links, and (g) proportions of links found using each process.

The processes used to identify links were categorised as one of three types: automatic, inferred, and inquired. *Automatic* links were defined by any process that used the unique registry identifier to reconcile the link into or from a bibliographic database without the need for a search or inquiry. This included searching PubMed for registry identifiers to find published articles in cohorts of registry entries or using identifiers in the metadata, abstract, or full text of published articles to find registry entries in cohorts of published articles. *Inferred* links were defined by any manual processes in which investigators searched for matches across databases using characteristics of the trial such as the names of the investigators, titles, and acronyms associated with the trial, location, sample size, or the population, intervention, or measurable outcome information to find a match in a bibliographic database or trial registry. *Inquired* links were defined by any manual process where the study authors attempted to contact the investigators or authors of a trial to request or confirm the presence or absence of a registry entry or a published article for each included trial.

### Data synthesis and analysis

We examined the proportions of links that were identified through each of these three processes. Using the publication year of the studies that used both automatic and manual processes, we applied linear regression to determine whether the utility of the automatic processes—the proportion that were found automatically compared to the proportion that required manual processes—had increased over time. We did not undertake a pooled analysis of the utility of automatic links because many studies did not specify proportions found by each process used and because of the heterogeneity in the study designs. All statistical analyses were conducted using SPSS statistical software version 24.0 (IBM, Armonk, NY).

The protocol for this systematic review was published in 2016 [22] (see Additional file 3). We did not register the systematic review with PROSPERO because it does not directly examine at least one outcome of direct patient or clinical relevance.

## Results

**Fig. 1:**
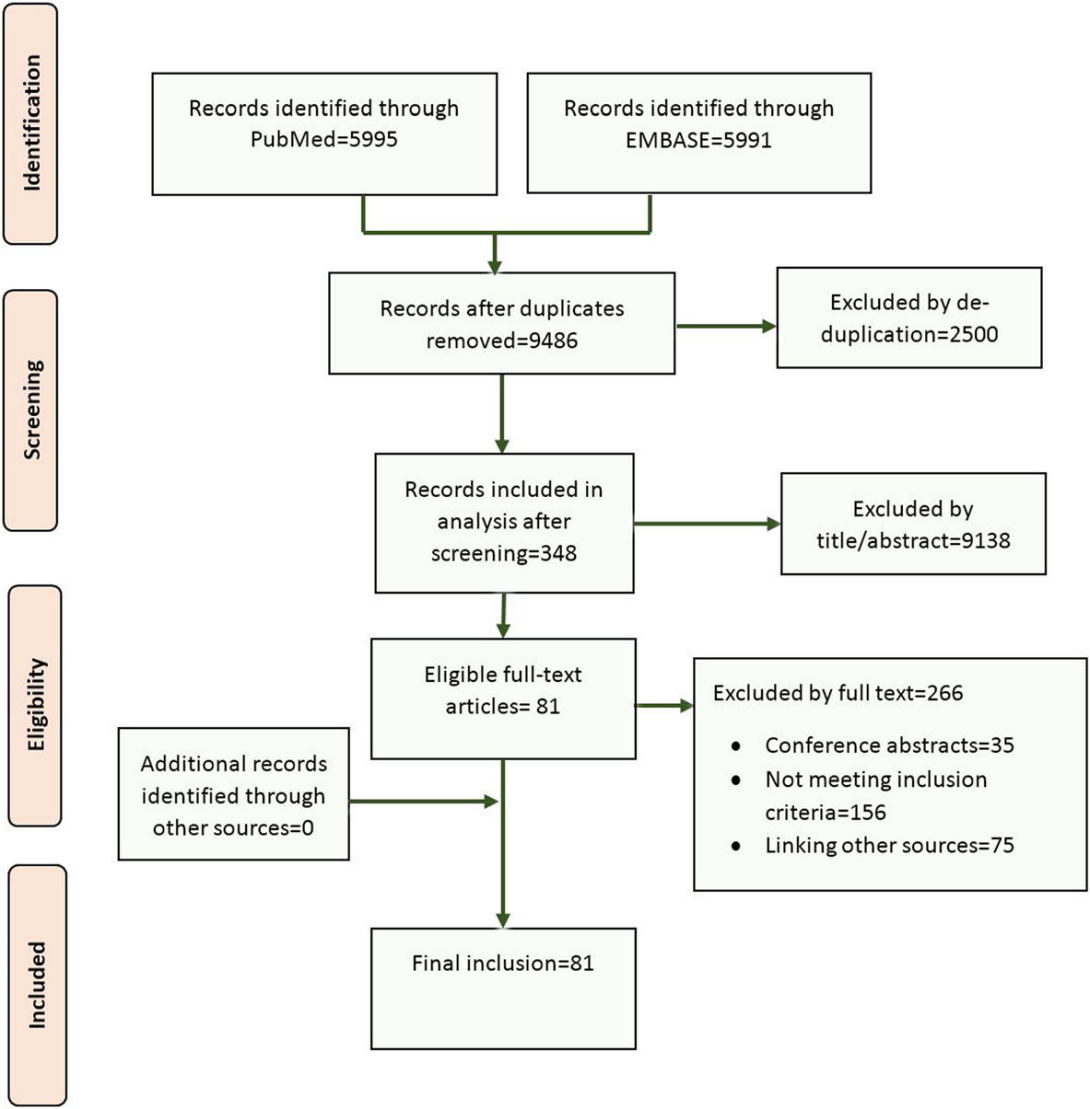
PRISMA flow diagram of study selection for a search and screening process that resulted in the inclusion of 81 studies

The initial search returned 11,986 results (after non-English articles were excluded), which produced 9486 articles after de-duplication (Fig. 1) [25]. A set of 348 studies remained after screening titles and abstracts, and of these, 81 studies were included in the review. One study considered links from both cohorts of registry entries and published articles [15, 26], for a total of 82 analyses. Excluded studies included conference abstracts, studies for which information about the proportions of registry entries or published articles that were identified was ambiguous [27–29] and studies that considered reporting biases but could not be included because the linking was atypical or there was no linking performed [30–33]. Some studies were excluded because they did not measure links between trial registries and bibliographic databases and, instead, considered links to or from other source of clinical trial information. These included links to or from protocols [34–37], conference or meeting abstracts [38–42], internal company documents [17], Food and Drug Administration (FDA) documents or new drug approvals [43–47], or other databases of published articles [48, 49].

### Studies identifying published articles from cohorts of registry entries

We identified 43 studies that examined links to published articles from registries, typically with the aim of examining publication bias or outcome reporting bias (Table 1). The application domains varied by types of studies (e.g., terminated and withdrawn trials [50, 51], trials funded by specific organisations or from certain countries [52, 53]), and by specialty and condition (e.g., paediatric or surgical trials [54, 55]). The most commonly studied registry was ClinicalTrials.gov only (35 studies), followed by some or all the registries of the WHO ICTRP (8 studies). The most commonly examined bibliographic databases were PubMed alone (22 studies), or Embase in combination with PubMed or other bibliographic databases (20 studies). The studies included cohorts of registry entries that ranged in size from 34 to 8907 (median 305) entries. The median proportion of registry entries for which published articles were found was 47%, and these proportions ranged from 4% (2 published articles in a cohort of 46 registry entries) to 76% (47 published articles in a cohort of 62 registry entries).

**Table 1:** Characteristics of 43 analyses identifying published articles from cohorts of trial registry entries

Study	Registry entry cohort	Published articles found	Trial registries included	Study purpose	Study publication year	Application domain	Proportion of links by process
Hartung [70]	305	110	ClinicalTrials.gov	To determine consistency between registered trials and their publication	2014	Phase III or IV trials	Automatic = 95 Inferred = 15
Ross [51]	677	315	ClinicalTrials.gov	To assess the publication of registered trials in ClinicalTrials.gov	2009	Completed trials of phase II or higher	Automatic = 96 Inferred = 215 Inquired = 4 (contact = 117, responded = 44, published = 4)
Bourgeois [71]	546	362	ClinicalTrials.gov	To determine whether funding source of these trials is associated with favorable published outcomes	2010	Anticholesteremics, antidepressants, antipsychotics, proton-pump inhibitors, and vasodilators	Inferred = unknown Inquired = unknown
Liu [72]	443	156	ANZCTR, ISRCTN, ChiCTR, IRCT, DRKS, NTR, JPRN, SLCTR, CTRI, PACTR, Clinicaltrials.gov.	Publication rate of Chinese Trials in WHO Registries	2010	Trials sponsored by China	Automatic = 103 Inferred = 40 Inquired = 13 (contact = 54, responded = all, published = 1)
Prenner [73]	64	35	Clinicaltrials.gov	To evaluate the rate of publication of registered clinical trials concerning age-related macular degeneration	2009	Muscular degeneration	Automatic = 8 Inferred = 27
Wildt [74]	105	66	ClinicalTrials.gov	To evaluate the adequacy of reporting of protocols for on diseases of the digestive system	2011	Gastrointestinal diseases	Inferred = 66
Gandhi [75]	37	20	ClinicalTrials.gov	To compare the published orthopaedic trauma trials following registration in ClinicalTrials.gov	2011	Orthopaedic trauma	Automatic and Inferred = unknown
Ross [53]	635	432	ClinicalTrials.gov	To review patterns of publication of clinical trials funded by NIH in peer reviewed biomedical journals	2012	NIH-funded trials in biomedical journals	Automatic and Inferred = unknown
Shamliyan [76]	758	212	ClinicalTrials.gov	To examine registration, completeness and publication of children studies	2012	Children studies funded by NIH	Inferred = 212
Vawdrey [77]	62	47	ClinicalTrials.gov	To measure the rate of non-publication and assess possible publication bias in clinical trials of electronic health records	2012	Electronic health record registered in clinicaltrials.gov	Automatic, inferred, and inquired = unknown
Chapman [55]	314	208	ClinicalTrials.gov	To determine the rate of early discontinuation and non-publication of RCTs	2014	Surgery	Inferred = 192 Inquired = 16 (contact = 101, responded = 25, published = 16)
Liu [52]	505	115	All 14 registries in ICTRP and ClinicalTrials.gov	To estimate bias risk and outcome-reporting bias in RCTs of traditional Chinese medicine	2013	Traditional Chinese medicines	Unknown
van de Wetering [26]	599	312	NTR	To evaluate the reporting of trial registration numbers in biomedical publications	2012	Biomedical publications	Automatic and inferred = unknown Inquired = 0 (contact = 42, responded = 9, published = 0)
Huser [15]	8907	885	ClinicalTrials.gov	Linking ClinicalTrials.gov with PubMed	2013	Interventional phase II or higher clinical trials	Automatic = 885
Stockmann [78]	108	65	ClinicalTrials.gov	To evaluate the publication patterns of obstetric studies registered in ClinicalTrials.gov	2014	Obstetric studies	Automatic = 45 Inferred = 20
Jones [79]	585	414	ClinicalTrials.gov	To estimate the frequency with which results of large randomized clinical trials registered with ClinicalTrials.gov are not available to the public	2013	Interventional RCTs with more than one arm	Automatic and inferred = unknown Inquired = 4
Riveros [80]	594	297	ClinicalTrials.gov	To assess timing and completeness of trial results posted at ClinicalTrials.gov and published in journals	2013	Interventional studies of phase III and IV	Unknown
Korevaar [81]	418	224	ClinicalTrials.gov	To assess publication and reporting of test accuracy studies registered in ClinicalTrials.gov	2014	Test accuracy studies	Automatic = 154 Inferred = 64 Inquired = 6 (contact = 175, responded = 119, published = 6)
Munch [82]	391	118	ICTRP, ClinicalTrials.gov	To analyse the perils and pitfalls of constructing a global open-access database of registered analgesic clinical trials	2014	Analgesic clinical trials	Inferred = 118
Hill [54]	90	66	ClinicalTrials.gov	To assess the characteristics of paediatric cardiovascular clinical trials registered on ClinicalTrials.gov	2014	Pediatric cardiovascular clinical trials	Unknown
Khan [83]	143	95	ClinicalTrials.gov	To examine characteristics associated with the publication and timeliness of publication of RCTs of treatment of rheumatoid arthritis	2014	Rheumatoid Arthritis	Automatic and inferred = unknown Inquired = 1 (contact = 58, responded = 28, published = 1)
Su [84]	239	88	All 14 registries in ICTRP and ClinicalTrials.gov	Outcome reporting bias	2015	Acupuncture	Automatic and inferred = unknown
Hakala [85]	177	102	ClinicalTrials.gov	To quantify the proportion of trials for unsuccessfully licensed drugs that are not published	2015	Stalled drugs	Automatic = unknown Inferred = unknown Inquired = 0 (emails or calls = 42, responded = 9, published = 0)
Pranic [50]	81	21	ClinicalTrials.gov	Outcome reporting bias	2016	Completed RCTs	Inferred = 21
Tang [86]	300	222	ClinicalTrials.gov	Outcome reporting bias	2015	Random sample of phase II or IV trials	Automatic and inferred = unknown
Boccia [87]	1109	120	ClinicalTrials.gov	To assess the status of registration of observational studies	2015	Cancer	Inferred = 120
Saito [88]	400	229	ClinicalTrials.gov	To determine publication rates of completed US trials	2014	Interventional studies	Automatic = 126 Inferred = 103
Son [89]	161	62	ClinicalTrials.gov	To assess whether there is publication bias in industry funded clinical trials of degenerative diseases of the spine	2015	Diseases of the spine	Inferred = 62
Baudart [90]	489	189	ClinicalTrials.gov	To evaluate the publication rate of observational studies for intervention	2016	Observational studies with safety outcomes	Automatic = 75 Inferred = 99 Inquired = 15 (contact = 241, responded = 52, published = 15)
Chahal [91]	34	20	ClinicalTrials.gov	To determine publication rates of RCTs in sports medicine	2012	Sports medicine	Automatic and Inferred = unknown
Manzoli [92]	355	176	ClinicalTrials.gov, ICTRP, ANZCTR, ChiCTR, Current Control Trails, Clinical Study Register or Indian	To evaluate the extent of non-publication or delayed publication of registered RCTs on vaccines	2014	Vaccines	Automatic = 132 Inferred = 44 Inquired = 0, (contact = 24, responded = 0, published = unknown)
Lebensburger [93]	147	52	ClinicalTrials.gov	To analyse ClinicalTrials.gov for registered sickle cell trials	2015	Sickle cells	Automatic = 28 Inferred = 24
Smith [94]	101	25	ClinicalTrials.gov	Outcome reporting bias	2012	Arthroplasty	Automatic = 10 Inferred = 15
Guo [95]	35	11	ClinicalTrials.gov	To estimate patterns of publication of clinical trials of endometriosis registered in ClinicalTrials.gov	2013	Endometriosis	Inquired = 8 Inferred = 3
Tsikkinis [96]	333	141	ClinicalTrials.gov	To identify all phase III prostate cancer trials in ClinicalTrials.gov with pending results	2015	Prostate cancer	Inferred = 141
Chen [97]	4347	2458	ClinicalTrials.gov	To assess publication rate and reporting of results for completed trials	2016	Interventional clinical trials	Automatic and inferred = unknown
Ramsey [98]	2028	357	ClinicalTrials.gov	To assess the proportion of registered trials that are published	2008	Oncology	Automatic = 357
Hurley [99]	142	62	ClinicalTrials.gov	To assess the delayed publication of clinical trials	2012	Cystic fibrosis	Inferred = 59 Inquired = 3 (contact = 83, responded = 29, published = 3)
Ioannidis [100]	73	21	Cochrane Controlled Clinical Trial Register, ISRCTN, ClinicalTrials.gov, ICTRP, GSK Clinical Study Register, and Indian, ANZCTR, and Chinese Clinical Trial Registries)	To assess publication delay	2011	Influenza A (H1N1) vaccination	Unknown
Ohnmeiss [101]	72	28	ClinicalTrials.gov	To assess the publication of the studies registered on ClinicalTrials.gov.	2015	Spine studies	Automatic and inferred = unknown
Gopal [102]	6251	818	ClinicalTrials.gov	To evaluated the rate of compliance with the FDA mandatory results reporting in clinicaltrials.gov	2012	Interventional studies	Automatic = 818
Lampert [103]	76	40	ClinicalTrials.gov	To determine selective outcome reporting and delay of publication	2015	Epilepsy	Automatic = 32 Inferred = 7 Inquired = 1
Gandhi [104]	46	2	ISRCTN, ClinicalTrials.gov, ANZCTR	To determine the extent to which ongoing and future RCTs in diabetes will ascertain patient-important outcomes	2008	Diabetes	Unknown

**Fig. 2:**
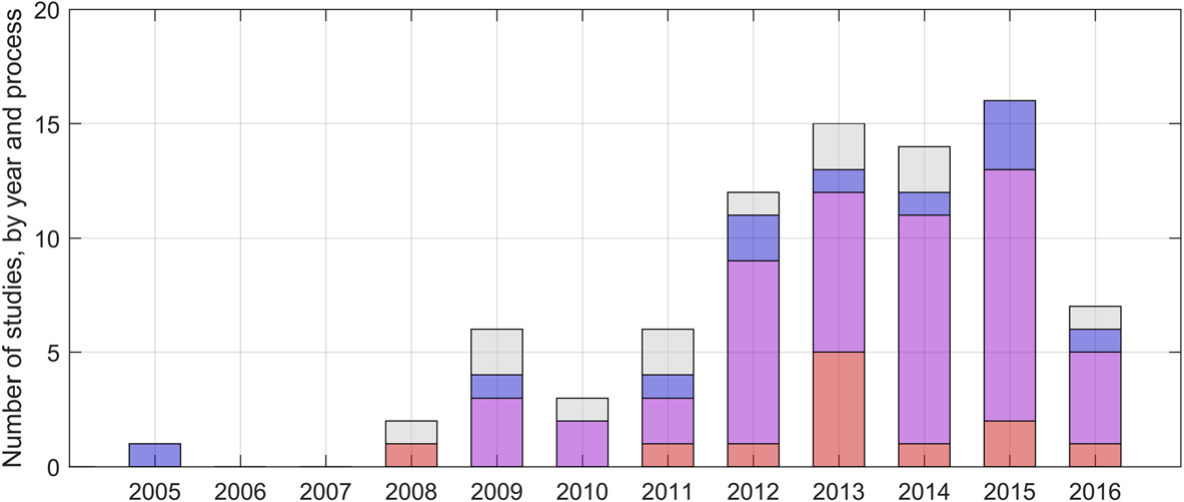
The processes used to identify links in 81 included studies, including studies that examined automatic links only (*red*), both automatic and manual processes (*purple*), manual processes only (*blue*), and studies that did not report the processes used (*grey*)

**Fig. 3:**
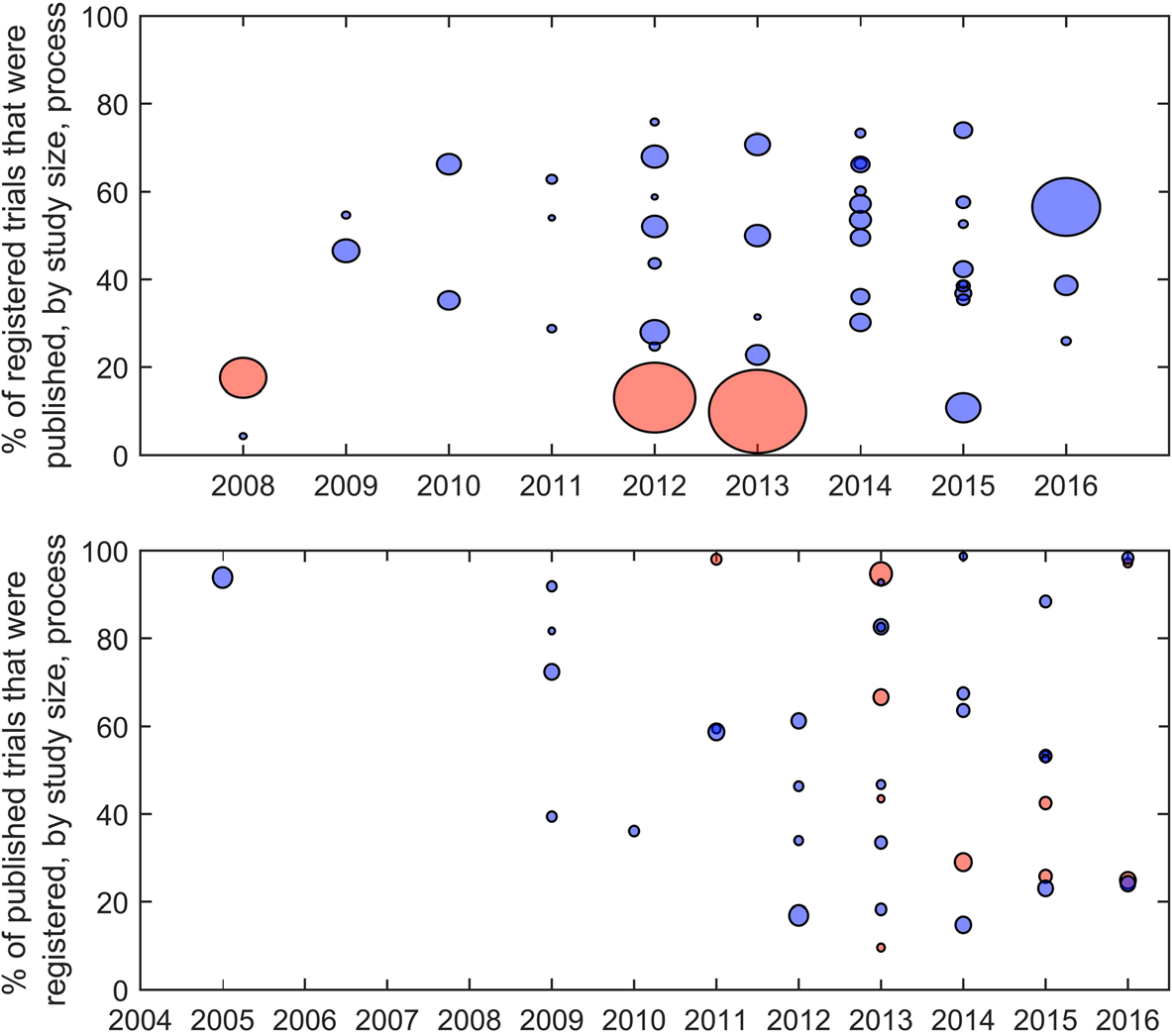
The proportions of published articles identified in cohorts of registry entries (*top*, 43 studies, ranging from 34 to 8907 registry entries) and the proportions of registry entries found in cohorts of published articles (*bottom*, 39 studies, ranging from 54 to 698 articles), with studies that only considered automatic links (*red*) and all other studies (*blue*). The circle areas are proportional to the study size

The processes used to identify links between clinical trial registries and published articles varied across the set of studies (Figs. 2 and 3). The most common process was to use a combination of automatic and manual processes (24/43, 56%), followed by manual processes only (11/43, 26%), and automatic processes only (3/43, 7%). There were five studies for which the process for identifying published articles was not clear or not provided.

**Fig. 4:**
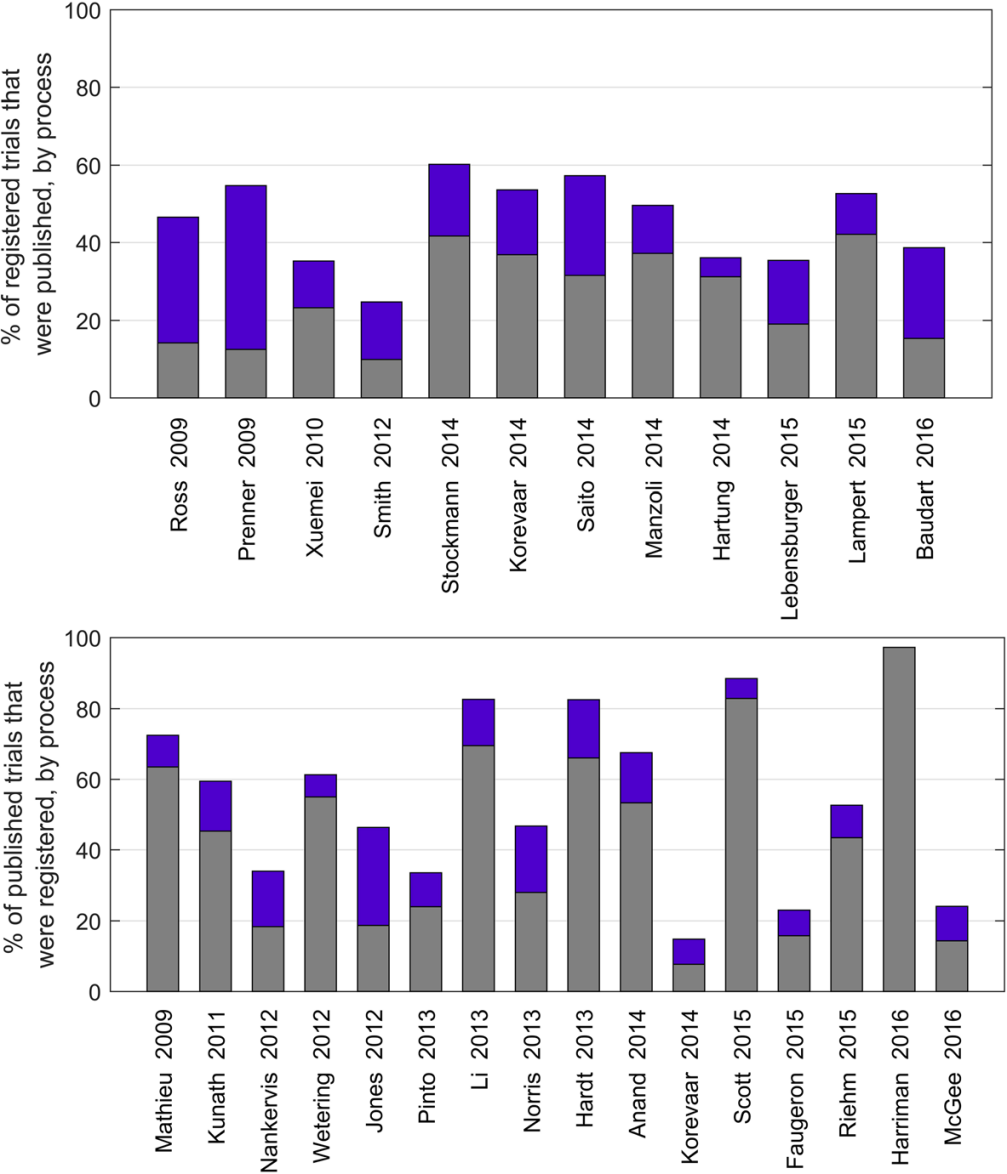
The proportions of published articles found in cohorts of registry entries (12 studies, *top*) and the proportions of registry entries found in cohorts of published articles (16 studies, *bottom*), by automatic links (*grey*) and manual processes (*blue*)

Of the 24 studies that looked for published articles among a cohort of registry entries and used both manual and automatic processes, 12 studies specified the number of published articles identified via each process (Fig. 4). Among these studies, automatic links were used to identify between 13 and 42% (median 23%) of the published articles, and manual processes were used to find a further 5–42% (median 17%) articles that were not available via automatic links.

We found no evidence of a change in the overall proportion of publications that could be found via automatic links. A linear regression over the 12 studies—using the publication year as the independent variable—indicated no significant trend in the proportion of available links that can be identified by automatic processes (*R* ^2^ = 0.02, *p* = 0.36, *β* = 1.28% increase per year).

### Studies identifying registry entries from cohorts of publications

There were 39 studies that considered cohorts of publications and identified associated registry entries in one or more of the WHO ICTRP clinical trial registries (Table 2). These studies included a range of 51–698 (median 181) published articles. These studies also covered a range of application domains, varying by the selection of journal, discipline, or study design [56–62]. The most commonly used bibliographic database was PubMed alone (19 studies), followed by PubMed in combination with other bibliographic databases (7 studies). To identify registrations, the studies most commonly searched ClinicalTrials.gov in combination with other registries (25 studies), followed by all trial registries included in the WHO ICTRP (9 studies). The median proportion of registry entries that were identified from cohorts of published articles was 54%, ranging from 10% (8 registrations from a cohort of 83 published articles) to 99% (75 registrations from a cohort of 76 published articles).

**Table 2:** Characteristics of 39 analyses identifying trial registry entries from cohorts of published articles

Study	Published article cohort	Registry entries found	Trial registries included	Study purpose	Study publication year	Application domain	Proportion of links by process
Mathieu [59]	234	323	ClinicalTrials.gov, ISRCTN, ICTRP, national register based on country of first author	Outcome reporting bias	2009	Cardiology, rheumatology, gastroenterology	Automatic = 205 Inferred = 6 Inquired = 23
Chowers [105]	49	60	Unknown	Outcome reporting bias	2009	Anti-retroviral therapy	Unknown
Rasmussen [60]	54	137	ClinicalTrials.gov, ISRCTN, ICTRP, NCI-PDQ	To determine association of trial registration with the results and conclusions of published trials	2009	Oncology drugs	Inferred = 54
Kunath [58]	63	106	ICTRP	To observe trial registration in urology journals	2011	Urology	Automatic = 48 Inferred = 15
Ewart [56]	135	124	ISRCTN, ClinicalTrials.gov, ANZCTR, EU-CTR, National Research Register	Outcome reporting bias	2009	RCTs in five high-impact factor journals	Unknown
You [106]	215	366	ClinicalTrials.gov, ISRCTN	Outcome reporting bias	2011	Oncology drugs	Unknown
Reveiz [109]	89	526	ICTRP	Outcome reporting bias	2012	RCT from Latin America and Caribbean	Unknown
Nankervis [108]	37	109	ICTRP	Outcome reporting bias	2012	Eczema treatment	Automatic = 20 Inferred = 17
Pinto [107]	67	200	ClinicalTrials.gov, ISRCTN, ANZCTR, national register based on country of first author	Completeness of clinical trial registration and the extent of selective reporting of outcomes in published trials	2013	Physical therapy	Automatic = 48 Inferred = 2 Inquired = 17
van de Wetering [26]	185	302	ClinicalTrials.gov, ISRCTN, ICTRP, national register based on country of first author	To determine reporting of trial registration numbers in biomedical publications	2012	RCT from core clinical journals	Automatic = 166 Inquired = 19 (contact = 136, responded = 51, published = 19)
Hannink [110]	218	327	ClinicalTrials.gov, ISRCTN, ANZCTR and others	Outcome reporting bias	2013	Surgical interventions	Automatic = 218
Huser [16]	661	698	ClinicalTrials.gov.gov, ISRCTN	Evaluating adherence to ICMJE policy of mandatory and timely clinical trial registration	2013	Trials published in five ICMJE journals	Automatic = 661
Rosenthal [111]	51	55	ClinicalTrials.gov, ISRCTN, ANZCTR, ChiCTR, UMIN	Outcome reporting bias	2013	Surgery	Automatic, inferred, and inquired = unknown
Hopewell [112]	30	69	Unknown	To observe reporting characteristics of non-primary publications of results of RCTs	2013	RCTs from National Library of Medicine’s set of 121 core clinical journals	Automatic = 30
Babu [113]	121	417	Unknown	To observe clinical trial registration in physical therapy journals	2014	Physical therapy journals	Automatic = 121
Lee [114]	8	83	Unknown	Assessment of compliance of randomized controlled trials in trauma surgery with the CONSORT statement	2013	Trauma surgery	Automatic = 8
Li [115]	252	305	ClinicalTrials.gov, Current Controlled Trials, NTR, ANZCTR, UMIN CTR	Outcome reporting bias	2013	Gastroenterology and herpetology	Automatic = 212 Inferred = 40
Norris [116]	50	107	ICTRP	To determine selective outcome reporting	2013	Pharmacotherapy	Automatic = 30 Inferred = 20
Hardt [117]	85	103	ICTRP(ClinicalTrials.gov, ISRCTN, EU-CTR, NTR, ANZCTR, DRKS, JPRNUMIN, ChiCTR, CTRI), Belgian register	To determine whether the results of registered surgical RCTs are published in journals requiring registration	2013	Ten highest rank surgery journals	Automatic = 68 Inferred = 17
Anand [118]	133	197	ClinicalTrials.gov, ISRCTN, ANZCTR	To determine the registration and design alterations of clinical trials in clinical care	2014	RCT in clinical care medicine	Automatic = 105 Inferred = 28
Mann [119]	140	220	ICTRP	To assess the registration status of RCTs and analyse the correspondence of registered outcomes with published outcomes	2014	Clinical geriatrics	Unknown
Walker [120]	75	76	ISRCTN, ClinicalTrials.gov, national register based on country of first author	Outcome reporting bias	2014	RCTs published in British Medical Journal and the Journal of American Medical Association	Automatic and inferred = unknown
Dekkers [121]	29	54	ICTRP	To compare non-inferiority margins defined in study protocols and trial registry records with margins reported in subsequent publications	2015	Non-inferiority trials submitted 2001–2005 to ethics committees in Switzerland and Netherlands	Automatic and inferred = unknown
Østervig [122]	85	200	ISRCTN, IRCT, EU-CTR, ChiCTR, CRiS, UMIN CTR, ClinicalTrials.gov	To check registration of randomized clinical trials	2015	Trials in Acta Anaesthesiologica Scandinavica	Automatic = 85
Scott [62]	160	181	ISRCTN, NTR, ANZCTR, ClinicalTrials.gov, national register based on country of first author	Selective outcome reporting	2015	Psychiatry journals	Automatic = 150 Inferred = 6 Inquired = 4
De Oliveira [123]	107	201	ISRCTN, ClinicalTrials.gov, ICTRP	Outcome reporting bias	2015	Anaesthesiology	Automatic, inferred, and inquired = unknown
Rayhill [61]	58	225	ClinicalTrials.gov and others	To assess the registration status of RCTs and analyse the correspondence of registered outcomes with published outcomes	2015	Core headache medicine journals	Automatic = 58
Dal-Ré [124]	175	178	ClinicalTrials.gov, ISRCTN, ANZCTR, NTR, EU-CTR, CTRI, DRKS	To evaluate adherence to ICMJE policy on prospective trial registration	2016	Trials in high-impact journals	Unknown
Reveiz [125]	52	144	Registered in any international clinical trial registry	To evaluate the influence of trial registration on reporting quality of RCTs	2010	Highest rank journals	Unknown
Rongen [126]	90	362	ClinicalTrials.gov, ISRCTN, ANZCTR, NTR and others	Outcome reporting bias	2016	Orthopedic surgical interventions	Automatic = 90
Harriman [57]	105	108	ClinicalTrials.gov, ISRCTN, ANZCTR, UMIN CTR, NTR, ChiCTR, IRCT	To assess trial registration, analysis of prospective versus retrospective registration	2016	Clinical trials published in the BMC series	Automatic = 105 Inquired = 0
Vera-Badillo [129]	30	164	ClinicalTrials.gov	Outcome reporting bias	2013	Breast cancer	Automatic and inferred = unknown
McGee [128]	74	307	ICTRP	To determine whether trial is registered and declared registration in the publication	2016	Kidney transplantation	Automatic = 44 Inferred = 30
Huić [127]	149	152	ClinicalTrials.gov	To determine completeness and outcome reporting bias	2011	RCTs published in ICMJE journals	Automatic = 149
Chan [34]	519	553	Unknown	Outcome reporting bias	2005	RCTs indexed in PubMed	Inferred and inquired = unknown
Korevaar [130]	52	351	ClinicalTrials.gov, ISRCTN, national register based on country of first author	To identify the proportion of articles for which the corresponding study had been registered	2014	Test accuracy studies	Automatic = 27 Inferred = 11 Inquired = 14 (contact = 324, responded = 187, published = 14)
Jones [131]	57	123	ClinicalTrials.gov, ISRCTN, ICTRP, national register based on country of first author	Outcome reporting bias	2012	Emergency	Automatic = 23 Inferred = 34
Smaïl-Faugeron [132]	73	317	ICTRP	To assess the registration rate of RCTs	2015	Oral health	Automatic = 50 Inferred = 23
Riehm [133]	40	76	ISRCTN, ClinicalTrials.gov, ICTRP	Outcome reporting bias	2015	Psychosomatic and behavioral health	Automatic = 33 Inferred = 7

The processes used to identify links between clinical trial registries and published articles varied across the set of studies (Figs. 2 and 3). The most common process was to use a combination of automatic and manual processes (21/39, 54%), followed by automatic processes only (9/39, 23%), and manual processes only (2/39, 5%). There were 7 studies for which the processes used to identify registry entries were not clear or not provided.

Of the 21 studies that looked for registry entries among a cohort of published articles and used both manual and automatic processes, 16 reported the number of registry entries found using each process (Fig. 4). Among these studies, automatic links identified between 8 and 97% (median 49%) of registry entries and the manual processes identified between 0 and 28% (median 10%) additional entries.

We found no evidence of a change in the overall proportion of published articles for which registry entries could be found via automatic links. A linear regression over the 16 studies—using the publication year as the independent variable—indicated no significant trend in the proportion of links that can be identified via automatic processes (*R* ^2^ = 0.01, *p* = 0.73, *β* = 1.40% increase per year).

## Discussion

In this systematic review, we found that investigators use both automatic and manual processes to link registry entries and publications and that automatic links could be used to identify some but not all links between registry entries and published articles. We found no evidence that the utility of automatic processes had increased over time.

To the best of our knowledge, no other systematic review has examined the utility of automatic links between trial registries and bibliographic databases. Previous studies that examined the availability of automatic links provided a broad analysis of automatic links made available through ClinicalTrials.gov and PubMed but did not systematically evaluate the proportion of links that could additionally be resolved using manual processes [15, 16, 63]. Other systematic reviews have examined reporting biases as a topic and included subsets of the studies we included [14, 64], but focused on publication rates and the completeness and consistency of outcome reporting, which we did not evaluate here. Our review adds to this area of research by compiling information about a broader group of studies and synthesising what is known about the utility of automatic links, and the need for supplementing automatic processes with manual processes, in studies that rely on links between trial registries and bibliographic databases.

### Implications

Our results indicate that automatic links alone are a useful but not sufficient process for measuring rates of registration and publication or associated biases. Relying on automatic links to draw conclusions about the rate of non-publication will likely over-estimate the rate of non-publication. When aiming to monitor compliance with prospective registration of clinical trials, or monitoring publication practices and patterns, the limits of automatic links should be considered.

In general, the proportion of links identified by automatic processes was lower in studies that started with a cohort of registry entries and aimed to identify published articles, compared to studies that started with a cohort of published articles, and aimed to identify registrations. This may be a consequence of journals that have not yet established standards for registration [65] or have not implemented standards for incorporating registry identifiers in the information they pass to bibliographic databases.

The results also have implications for systematic reviews. Systematic review technologies for automating or supporting reviewers rarely consider information from clinical trial registries to improve the searching or screening processes [66] or the prioritisation or scheduling of systematic review updates. Because systematic reviews are already time-consuming [67, 68], the need for additional manual effort in the linking of trial registry entries with their published results may have hindered the development of tools based on this linkage. Areas for development include processes where systematic reviewers compare published reports with information in a registry or use trial registries to identify trials not found in bibliographic databases. By removing these barriers, machine-readable information linking all published studies with all registry entries may provide the catalyst for the increased use of registries in the searching, screening, and prioritising of systematic reviews.

### Recommendations

We recommend continued pressure to ensure that journals and publishers adhere to standards of reporting that require unique trial identifiers to be specified in the abstract of the article and reported as part of the metadata provided to bibliographic databases. Trial investigators should also be encouraged to update registry entries with links to published results when journals do not provide the information to bibliographic databases. As we move into an era where the structured reporting of clinical trial results and individual participant data become the standard for responsible clinical trial reporting [69], the inability to automatically identify all sources of information about a clinical trial hinders our ability to reuse and synthesise results across trials. Given the number of extra links that could be identified by examining the full text of articles, we also recommend that journals ensure that clinical trial identifiers are included in the abstract or metadata provided to bibliographic databases.

We additionally recommend a standardised method for identifying links between registry entries and published articles that, for the time being, includes manual validation and checking and avoids drawing conclusions based only on automatic links. A standardised method should include details about what elements of a registry entry should be used to search for published articles and a standard definition for what constitutes published results. Standard reporting for these studies should include the number of registry entries for which searches were performed, the proportion that were identified by automatic links, by inference or by inquiry, and the full details of the dates of trial completion and the length of follow-up. Presenting studies in terms of the time to publication rather than the presence or absence of publication would make a greater proportion of the studies comparable and amenable to meta-analysis.

### Limitations

There are two limitations to this review. First, the exclusion of studies for which there was no English language version available meant that we may have missed some studies examining WHO ICTRP registries from countries where English is not the primary language. Second, we used the publication year of the studies as a proxy for estimating changes in the proportions of links identified by each process without considering the period of study that each of the studies covered. This was necessary because a substantial proportion of studies did not report the range and distribution of publication and registration dates in the cohorts they examined, and this may have influenced our analysis of the trends in the utility of the automatic processes.

## Conclusions

In this systematic review, we have quantified the use and utility of the processes that are used to link trial registries to bibliographic databases. The results indicate that manual processes are still used extensively and that the gap between what can be identified via automatic processes and what must be identified via manual processes persists. Future improvements in the quality of automatic linking between clinical trial registries and bibliographic databases should come from continued pressure on journals to enforce policies and practices to consistently include registry identifiers in published reports.

## Additional files

Additional file 1: Search strategy for PubMed. Search strategy for MEDLINE via PubMed. (PDF 259 kb)

Additional file 2: Search strategy for Embase. Search strategy for Embase via Ovid. (PDF 309 kb)

Additional file 3: PRISMA checklist. PRISMA Checklists with manuscript page number reference. (PDF 277 kb)

## Abbreviations

ICTRP: International Clinical Trial Registry Platform
WHO: World Health Organization
