# Supplementary material for: A systematic review of the processes used to link clinical trial registrations to their published results
Source: Syst Rev. 2017 Jul 3;6:123. doi: 10.1186/s13643-017-0518-3 (PMC5494826; doi:10.1186/s13643-017-0518-3)
Supplement: Supplementary file 1 — Search strategy for PubMed. Search strategy for MEDLINE via PubMed. (PDF 259 kb) [file 13643_2017_518_MOESM1_ESM.pdf]

Additional file 1. Search strategy for MEDLINE via PubMed

	Search Terms
#1	clinicaltrials.gov[Title/Abstract]
#2	ANZCTR[Title/Abstract]
#3	ICTRP[Title/Abstract]
#4	ReBec[Title/Abstract]
#5	ChiCTR[Title/Abstract]
#6	CRiS[Title/Abstract]
#7	CTRI[Title/Abstract]
#8	RPCEC[Title/Abstract]
#9	EU-CTR[Title/Abstract]
#10	DRKS[Title/Abstract]
#11	IRCT[Title/Abstract]
#12	JPRN[Title/Abstract]
#13	NTR[Title/Abstract]
#14	ISRCTN[Title/Abstract]
#15	PACTR[Title/Abstract]
#16	SLCTR[Title/Abstract]
#17	trial registry"[Title/Abstract]
#18	"trial register"[Title/Abstract]
#19	"trial registries"[Title/Abstract]
#20	"trials registry" [Title/Abstract]
#21	"registry of clinical trials" [Title/Abstract])]
#22	#1 or #2 or #3 or #4 or #5 or #6 or #7 or #8 or #9 or #10 or #11 or #12 or #13 or #14 or #15 or #16 or #17 or #18 or #19 or #20 or #21
#23	"trial registration"[Title/Abstract]
#24	discrepancy[Title/Abstract]
#25	discrepancies[Title/Abstract]
#26	consistency[Title/Abstract]
#27	inconsistency[Title/Abstract]
#28	#24 or #25 or #26 or #27
#29	#23 and #28
#30	#22 or #29
#31	unregistered[Title/Abstract]
#32	non-publication[Title/Abstract]
#33	nonpublication[Title/Abstract]
#34	unpublished[Title/Abstract]
#35	published[Title/Abstract]
#36	registered[Title/Abstract]
#37	#31 or #32 or #33 or #34 or #35 or #36
#38	publication[Title/Abstract]
#39	clinical trial as topic [MeSH Terms]
#40	#38 or #39
#41	#37 and #40
#42	#30 and #41
#43	"outcome reporting bias"[Title/Abstract]
#44	"selective reporting"[Title/Abstract]
#45	"selective outcome reporting"[Title/Abstract]
#46	"missing outcome data"[Title/Abstract]
#47	"publication bias"[MeSH Terms]
#48	#43 or #44 or #45 or #46 or #47
#49	"reporting quality"[Title/Abstract]
#50	publications[Title/Abstract]
#51	#49 and #50
#52	#48 or #51
#53	#42 or #52
