# Supplementary material for: A systematic review of the processes used to link clinical trial registrations to their published results
Source: Syst Rev. 2017 Jul 3;6:123. doi: 10.1186/s13643-017-0518-3 (PMC5494826; doi:10.1186/s13643-017-0518-3)
Supplement: Supplementary file 2 — Search strategy for Embase. Search strategy for Embase via Ovid. (PDF 309 kb) [file 13643_2017_518_MOESM2_ESM.pdf]

Additional file 2. Search strategy for EMBASE via Ovid.

	Search Terms
#1	"clinicaltrials.gov:".tw
#2	ANZCTR:.tw.
#3	ICTRP:.tw.
#4	ReBec:.tw.
#5	ChiCTR:.tw.
#6	CRiS:.tw.
#7	CTRI:.tw.
#8	RPCEC:.tw
#9	EU-CTR:.tw.
#10	DRKS:.tw.
#11	IRCT:.tw.
#12	NTR:.tw.
#13	ISRCTN:.tw.
#14	PACTR:.tw.
#15	SLCTR:.tw.
#16	JPRN:.tw.
#17	"trial registry:".tw.
#18	"trial register:".tw.
#19	"trial registries:".tw.
#20	"trials registry:".tw.
#21	"registry of clinical trials:".tw.
#22	#1 or #2 or #3 or #4 or #5 or #6 or #7 or #8 or #9 or #10 or #11 or #12 or #13 or #14 or #15 or #16 or #17 or #18 or #19 or #20 or #21
#23	"trial registration:".tw.
#24	discrepancy:.tw.
#25	discrepancies:.tw.
#26	consistency:.tw.
#27	inconsistency:.tw.
#28	#24 or #25 or #26 or #27
#29	#23 and #28
#30	#22 or #29
#31	unregistered:.tw.
#32	non-publication:.tw.
#33	nonpublication:.tw.
#34	unpublished:.tw.
#35	published:.tw.
#36	#31 or #32 or #33 or #34 or #35
#37	registered:.tw.
#38	publication:.tw.
#39	"clinical trial (topic)"/
#40	#38 or #39
#41	#37 and #40
#42	#36 or #41
#43	#30 and #42
#44	"outcome reporting bias:".tw.
#45	"selective reporting:".tw.
#46	"selective outcome reporting:".tw.
#47	"missing outcome data:".tw.
#48	#44 or #45 or #46 or #47
#49	"reporting quality:".tw.
#50	publications:.tw.
#51	#49 and #50
#52	#48 or #51
#53	#43 or #52
